# A novel targeted RNA-Seq panel identifies a subset of adult patients with acute lymphoblastic leukemia with *BCR-ABL1*-like characteristics

**DOI:** 10.1038/s41408-020-0308-3

**Published:** 2020-04-24

**Authors:** Ricardo Sánchez, Jordi Ribera, Mireia Morgades, Rosa Ayala, Esther Onecha, Yanira Ruiz-Heredia, Alexandra Juárez-Rufián, Rodrigo de Nicolás, José Sánchez-Pina, Susana Vives, Lurdes Zamora, Santiago Mercadal, Rosa Coll, Marta Cervera, Olga García, Josep-Maria Ribera, Joaquín Martínez-López

**Affiliations:** 1 Hematology Department, Hospital Universitario 12 de Octubre, Madrid, Spain; 2 Instituto de Investigación Hospital 12 Octubre (i+12), Madrid, Spain; 3 Haematological Malignancies Clinical Research Unit, Spanish National Cancer Research Centre (CNIO), Madrid, Spain; 4 Hematology Department, ICO – Hospital Germans Trias i Pujol. Josep Carreras Leukaemia Research Institute, Universitat Autònoma de Barcelona, Badalona, Spain; 5 Centro de Investigación Biomédica en Red Cáncer (CIBERONC), Madrid, Spain; 6 Universidad Complutense de Madrid, Madrid, Spain; 7 Hematology Department, ICO – Hospital Duran i Reynals (Bellvitge), Barcelona, Spain; 8 Hematology Department, ICO – Hospital Dr. Josep Trueta, Girona, Spain; 9 Hematology Department, ICO – Hospital Universitari Joan XXIII, Tarragona, Spain

**Keywords:** Translational research, Cancer genomics, Genetics research

## Abstract

*BCR-ABL1*-like B-cell precursor acute lymphoblastic leukemia (BCP-ALL) remains poorly characterized in adults. We sought to establish the frequency and outcome of adolescent and adult *BCR-ABL1*-like ALL using a novel RNA-Seq signature in a series of patients with BCP-ALL. To this end, we developed and tested an RNA-Seq custom panel of 42 genes related to a *BCR-ABL1*-like signature in a cohort of 100 patients with BCP-ALL and treated with risk-adapted ALL trials. Mutations related to *BCR-ABL1*-like ALL were studied in a panel of 33 genes by next-generation sequencing (NGS). Also, *CRLF2* overexpression and *IKZF1*/*CDKN2A/B* deletions were analyzed. Twenty out of 79 patients (12–84 years) were classified as *BCR-ABL1*-like (25%) based on heatmap clustering, with significant overexpression of *ENAM*, *IGJ*, and *CRLF2* (*P* ≤ 0.001). The *BCR-ABL1*-like subgroup accounted for 29% of 15–60-year-old patients, with the following molecular characteristics: *CRLF2* overexpression (75% of cases), *IKZF1* deletions (64%), *CDKN2A/B* deletions (57%), and *JAK2* mutations (57%). Among patients with postinduction negative minimal residual disease, those with the *BCR-ABL1*-like ALL signature had a higher rate of relapse and lower complete response duration than non-*BCR-ABL1*-like patients (*P* = 0.007). Thus, we have identified a new molecular signature of *BCR-ABL1*-like ALL that correlates with adverse prognosis in adult patients with ALL.

## Introduction

The 2016 World Health Organization (WHO)classification of acute leukemias recognizes nine different entities within B-cell precursor acute lymphoblastic leukemia/lymphoma (BCP-ALL) and two new provisional entities, including *BCR-ABL1*-like. These 11 subtypes are based on specific molecular alterations, mainly chromosome rearrangements, that promote the formation of aberrant chimeric proteins and aneuploidies^1^. Next-generation sequencing (NGS) and array technologies have been instrumental in identifying new ALL subtypes, and have aided in the discovery of new leukemogenic mechanisms. It has been known for many years that a subset of patients with ALL (~25% of BCP-ALL) have no established abnormalities, commonly referred to as B-other ALL. The genomic landscape of B-other ALL is becoming increasingly clear, and the proportion of unclassifiable patients has declined significantly^2,3^. In this context, *BCR-ABL1*-like B-ALL has emerged as one of the most relevant new subtypes due to its frequency and the potential benefit of targeted therapies (i.e., ABL and JAK inhibitors).

Philadelphia chromosome-positive ALL is defined by the t(9;22)(q34;q11) translocation that encodes *BCR-ABL1* oncogene, a constitutively active kinase. This aberration is present in >95% of patients with chronic myeloid leukemia, and in 3–5% and 25% of pediatric and adult ALL cases, respectively. In 2009, the DCOG/Erasmus and COG/St. Jude groups independently discovered a high risk *BCR-ABL1*-negative subgroup in children with B-ALL, exhibiting a gene expression signature similar to that of *BCR-ABL1* positive-ALL but lacking the *BCR-ABL1* rearrangement^4^. This subtype is associated with downregulation of B-cell development genes and overexpression of stem- and progenitor-cell genes. Clinically, this ALL subtype presents with high-risk clinical features such as high white blood cell (WBC) count, poor response to induction chemotherapy, higher measurable residual disease levels, and low probability of survival^4–8^.

The frequency of *BCR-ABL1*-like ALL has been reported as 20–30% in adults, with a peak of incidence in the adolescent and young adult population (up to 42%)^9–12^. The *BCR-ABL1*-like ALL subtype shows deletions in several transcription factors involved in B-cell development, including IKZF1, E2A, EBF1, and PAX5^13^. Additionally, the main molecular characteristics of *BCR-ABL1*-like ALL are the multiple translocations in different cytokine receptor and kinase signaling genes such as *ABL1* (excluding BCR association), *JAK2*, *ABL2*, *PDGFRB*, *TYK2*, *CSF1R*, *CRLF2*, and *EPOR*. These mutations trigger the activation of growth promoting kinase or cytokine signaling pathways^14^. *CRLF2* translocation and mutations in the JAK family genes are recurrent and result in the activation of JAK-STAT pathways in patients with *BCR-ABL1*-like B-ALL^15–17^.

Several different approaches have been employed for the characterization of *BCR-ABL1*-like ALL. Patients were classified using hierarchical clustering on a gene expression array in early studies^6,8^. A simpler approach was subsequently optimized based on Low Density Arrays carrying a small number of genes selected by microarray prediction analysis^15,18^. In the present study, we designed a targeted NGS RNA-Seq panel of 42 genes to classify patients with BCR*-ABL1*-like ALL. We sought to identify the *BCR-ABL1*-like ALL signature by targeted expression in a series of adolescent and adult patients with BCP-ALL, but without recurrent genetic abnormalities defined by the WHO classification (henceforth B-other ALL), and to evaluate its clinical, prognostic and therapeutic relevance.

## Patients and methods

### Patients and study design

We examined bone marrow (BM) or peripheral blood (PB) samples from adolescent and adult patients newly diagnosed with B-other ALL and treated between 2003 and 2017 in several Spanish hospitals. Patients received frontline chemotherapy according to PETHEMA (*Programa Español de Tratamientos en Hematología*) ALL risk and minimal residual disease (MRD)-oriented trials^19,20^. A first series of patients treated between 2002 and 2012 was evaluated for the identification of the *BCR-ABL1*-like signature (*n* = 49), and a second series of patients treated between 2012 and 2017 was used for validation (*n* = 100).

**Fig. 1:**
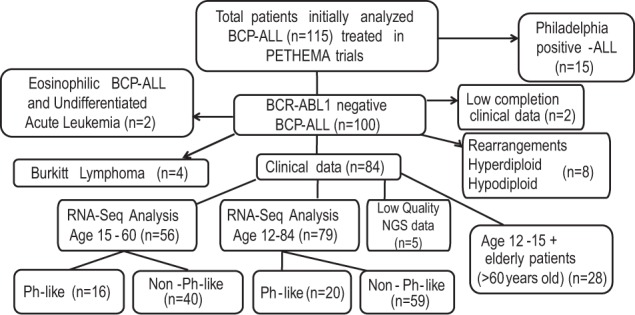
Study flow chart. Design and distribution of the patients. BCP-ALL: B-cell precursor acute lymphoblastic leukemia; NGS: Next-generation sequencing; Ph: *BCR-ABL1*.

The study design is shown in Fig. 1. One hundred patients with *BCR-ABL1*-negative B-ALL were selected for molecular studies. Of those, 16 were discarded [Burkitt Lymphoma (*n* = 4), undifferentiated acute leukemia (*n* = 2), ALL with MLL rearrangements (*n* = 2), *TCF3-PBX1* (*n* = 1), *ETV6/RUNX1* (*n* = 1), hyperdiploidy (*n* = 3) and hypodiploidy (*n* = 1) not classified as *BCR-ABL1*-like based on the RNA-Seq signature, and two patients with low quality clinical data]. The remaining 84 patients, classified as B-other ALL, were considered valid for *BCR-ABL1*-like signature identification by RNA-Seq. Of these, 28 were discarded for survival analysis due to the heterogeneity of treatment protocols (including palliative therapy) and the absence of MRD assessment. Therefore, the prognostic relevance of the *BCR-ABL1*-like signature was investigated in a cohort of 56 homogeneously treated patients (median age 34 years; range 16–59 years). The main clinical characteristics and prognosis of patients with *BCR-ABL1*-like B-ALL are shown in Table 1.

**Table 1:** Summary of clinical characteristics of the patients included in the study.

	Whole series (*N* = 56)	*BCR-ABL1*-like ALL (*n* = 16)	Remaining B-ALL (*n* = 40)	*P*-value
Age				
Median [range]	34 [16; 59]	22 [17; 59]	38.5 [16; 58]	0.310
Age range (years)				
15–35	30 (54%)	11 (69%)	19 (47%)	0.150
36–59	26 (46%)	5 (31%)	21 (53%)	
Sex				
Male	27 (48%)	10 (63%)	17 (42%)	0.176
Female	29 (52%)	6 (37%)	23 (58%)	
WBC·10^−3^/µL (*n* = 52)				
Median (range)	7.87 [0.60-393.30]	13.40 [1.90-388]	5.01 [0.60-393.30]	0.071
*CRLF2*/*GAPDH*				
Negative (≤0,1%)	35 (63%)	4 (25%)	31 (78%)	**<0.001**
Positive (>0,1%)	21 (37%)	12 (75%)	9 (22%)	
PB Blasts (%) (*n* = 56)				
Median [range]	41 [0; 95]	72 [24; 95]	34 [0; 90]	**0.002**
BM Blasts (%)(*n* = 30)				
Median [range]	93 [60; 100]	96,5 [93; 98]	92 [60; 100]	0.069
Phenotype				
Common	37 (66%)	10 (63%)	27 (68%)	0.417
Pre-B	12 (21%)	5 (31%)	7 (17%)	
Pro-B	7 (13%)	1 (6%)	6 (15%)	
Karyotype (*n* = 49; *n* = 15 *BCR-ABL1*-like; *n* = 34 remaining B-ALL)				
Normal	12 (25%)	3 (20%)	9 (27%)	0.428
IGHr	4 (8%)	1 (7%)	3 (9%)	
Complex^a^	2 (4%)	0 (0%)	2 (6%)	
No Growth	14 (29%)	3 (20%)	11 (32%)	
Other^b^	17 (35%)	8 (53%)	9 (27%)	
Risk classification				
High	46 (82%)	13 (81%)	33 (83%)	1.000
Intermediate	10 (18%)	3 (19%)	7 (17%)	
MRD^c^ postinduction (*n* = 41; *BCR-ABL1*-like *n* = 11; Remaining B-ALL *n* = 30)				
Positive	18 (44%)	8 (73%)	10 (33%)	**0.036**
Negative	23 (56%)	3 (27%)	20 (67%)	
MRD^c^ post-consolidation (*n* = 36; *BCR-ABL1*-like *n* = 8; Remaining B-ALL *n* = 28)				
Positive	10 (28%)	2 (25%)	8 (29%)	1.000
Negative	28 (72%)	6 (75%)	20 (71%)	
*IKZF1* gene deletion^d^				
Positive	23 (52%)	9 (64%)	14 (47%)	0.276
Negative	21 (48%)	5 (36%)	16 (53%)	
*CDKN2A/B* gene deletion^d^				
Positive	19 (43%)	8 (57%)	11 (37%)	0.202
Negative	25 (57%)	6 (43%)	19 (63%)	
*IKZF1* and *CDKN2A/B* codeletion^d^				
Positive	11 (25%)	5 (36%)	6 (20%)	0.287
Negative	33 (75%)	9 (64%)	24 (80%)	

*BM* bone marrow, *IGHr* rearrangements of IGH@ gene, *MRD* minimal residual disease, *PB* peripheral blood, *WBC* white blood cells.

^a^Complex karyotype is defined as more than four chromosomic alterations.

^b^Other: del 12p (*n* = 1); del6q (*n* = 1); del7p plus del9p (*n* = 1); del9p (*n* = 2); del6q plus del 12p (*n* = 1); other deletions (*n* = 3); other rearrangements (*n* = 4); other alterations (*n* = 4).

^c^Positive MRD ≥ 0.01 by multiparametric flow cytometry.

^d^*IKZF1* and *CDKN2A/B* deletions *n* = 44; *BCR-ABL1*-like *n* = 14; remaining B-ALL *n* = 30.

Details for patient samples are provided in Supplementary Table S1. The study was conducted in accordance with the principles of the Declaration of Helsinki, and the protocols were approved by the appropriate institutional review boards. All patients provided written informed consent for the analysis of their biological specimens.

### MRD assessment by multiparametric flow cytometry

BM MRD levels were centrally assessed at the end of induction (weeks 5–6) in complete remission (CR) patients and at the end of the third consolidation cycle (weeks 16–18) by multiparameter flow cytometry. MRD levels at this latter time point were used to assign post-consolidation therapy (continuation chemotherapy or allogeneic hematopoietic stem cell transplantation). The detection limit of the method was 1 × 10^−4^. MRD level was considered positive when exceeded 0.01% or 1 × 10^−4^ at the end of induction and after consolidation.

### Molecular biology analyses

#### *CRLF2* overexpression

One microgram of RNA was retrotranscribed to evaluate the expression levels of *CRLF2* relative to *GAPDH*, by real-time quantitative PCR (qPCR). The probes were acquired from Gene Expression Taqman Assays (Thermo Fisher, Palo Alto, CA): Hs00845692_m1 (*CRLF2*), and Hs02786624_g1 (*GAPDH*). Overexpression was determined by means of 2^−ΔΔCt^ method^21^, and was defined as positive when it was ≥0.1% relative to *GAPDH* gene expression.

#### Multiplex ligation-dependent probe amplification (MLPA)

One hundred ng of DNA was used for MLPA according to manufacturer instructions (MRC Holland, Amsterdam, the Netherlands). Two different kits have been used, one of them SALSA MLPA P335-C1 ALL-IKZF1 probemix kit contains 57 MLPA probes with amplification products between 120 and 504 nucleotides of the main exons of *EBF1, JAK2, CDKN2A, CDKN2B, PAX5, ETV6, BTG1, RB1, IKZF1/2/3* genes, and genes of the X/Y PAR1 region (*CRLF2, CSF2RA, IL3RA* and *P2RY8*). The second Kit SALSA MLPA P202 probemix kit contains 59 MLPA probes with amplification products between 118 and 504 nucleotides, which span *IKZF1* and *CDKN2A-B* genes (21 and 3 probes, respectively).

#### Targeted RNA-sequencing

cDNA was obtained after reverse transcription of 1 µg of RNA. cDNA integrity was checked by qPCR with a *GUSB* Taqman probe (Hs00939627_m1) (Thermo Fisher Scientific), discarding cDNA with a Ct>25 at a threshold of 0.1. A barcoded cDNA library was then generated by amplification using Ion AmpliSeq® (Thermo Fisher Scientific) technology to precisely maintain expression levels of the targeted genes. A targeted RNA-Seq customized panel was designed with 38 genes plus 4 housekeeping genes, generating 42 primer pair amplicons. The tested genes were as follows (in alphabetical order): *ABCA9, ARHGEF12, BMPR1B, CA6, CD99, CHN2, CRLF2, DCTN4, DENND3, ECM1, ENAM, GAB1, GBP5, GPR110, IFITM1, IGJ, IL7R, LDB3, MDFIC, MMP28, MUC4, NRXN3, PON2, RBM47, RNF157, S100Z, SEMA6A, SCHIP1, SH2B3, SLC2A5, SLC37A3, SOC2, SPATS2L, TAF5L, TMEM154, TP53INP1, TTYH2*, and *WNT9A*^22^. The control genes were *GUSB, JUN, PBGD*, and *TBP*. Two of the 42 genes (*SCHIP1* and *IFITM1*) were discarded from analysis due to the low number of reads in each of the runs.

The quality of the amplified cDNA libraries was evaluated using Bioanalyzer High Sensitivity chips (Agilent Technologies, Santa Clara, CA) and quantified with Ion Library TaqMan™ Quantitation Kits (Thermo Fisher Scientific). Libraries were diluted to 100 pM and pooled equally, assigning 150,000 reads per sample. Pooled libraries were amplified using the Ion Chef System with the Ion 540 Sequencing Kit (Thermo Fisher Scientific). Enriched libraries on a chip were sequenced on the Ion GeneStudio S5 System using the Ion S5 Sequencing Kit (Thermo Fisher Scientific) with 500 flows. The absolute normalized Reads Per Million (RPM) matrix was obtained from RNA-Seq Analysis plug-in (v5.4.0.1) within Torrent Suite software (v5.10) (Thermo Fisher Scientific). The matrix was subsequently normalized intra-patient relative to *GUSB* because it was the housekeeping gene with the higher Spearman correlation coefficient (ρ) of the reads between patients within the matrix data, compared to the other three genes. A final analysis was performed using the web-based tool Morpheus (https://software.broadinstitute.org/morpheus/), a matrix visualization and analysis platform, obtaining an unsupervised hierarchical cluster heatmap using one minus Pearson correlation as a metric, and an average of the data as the linkage method. The raw RNA-Seq sequencing data were uploaded to NCBI with BioProject ID: PRJNA613841.

#### Targeted DNA-sequencing

A total of 33 lymphoid-related genes were sequenced by targeted NGS using an Ion Ampliseq® On Demand panel (Thermo Fisher Scientific), consisting of a custom mixture of oligonucleotides that generated 892 amplicons in two pools, covering 182 kb. The design includes whole exons of *JAK1, NRAS, XPO1, CXCR4, SF3B1, MYD88, KLHL6, WHSC1, FBXW7, IRF4, IKZF1, CRLF2, BRAF, EZH2, JAK2, CDKN2A, PAX5, NOTCH1, ATM, KRAS, KMT2D, CREBBP, TP53, STAT5B, STAT3, TYK2*, and *JAK3* genes, and selected exons of *RAB39A, CUL5, EXPH5, DLEU1, SAT2*, and *EFNB3*.

Libraries were prepared following the Ampliseq® protocol using at least 10 ng of template DNA per reaction. Multiple indexed libraries were pooled and sequenced on the Ion GeneStudio S5 System using Ion S5 Sequencing Kit, with 500 flows. Samples were sequenced to an average 1900× coverage. Alignment and variant detection were performed using Ion Reporter v5.10 using the human reference genome (hg19). Variants were manually reviewed in Integrated Genome Viewer v2.3.81 (Broad Institute, Cambridge, MA). Variants were classified as benign, unknown significance, pathogenic or likely pathogenic according to VARSOME software^23^. The raw DNA-Seq sequencing data were uploaded to NCBI with BioProject ID: PRJNA614523.

#### Statistical analyses

Baseline characteristics were reported as frequency and percentage for categorical variables and as median and range for quantitative variables. Comparisons of proportions and the medians of variables between different groups were performed using the χ^2^ test, Fisher’s exact test, or the nonparametric median test as appropriate. Overall survival (OS) was measured from the time of diagnosis to the time of death from any cause. Disease-free survival (DFS) was measured from the date of achievement of the first remission until the date of relapse or death from any cause. Cumulative incidence of relapse (CIR) was calculated from the date of achievement of the first remission until the date of relapse. Patients who died without relapse were counted as a competing risk. Patients not known to have relapsed ordied at last follow-up were censored on the date they were last examined. OS and DFS curves were performed using the Kaplan–Meier estimation, and the log-rank test was used for comparisons between groups. CIR curves were estimated using cumulative incidence rates and were compared by Gray’s test. Two-sided *P*-values < 0.05 were considered statistically significant. Multivariable analyses were performed using the Cox proportional hazards model for OS and DFS, and the Fine and Gray model for CIR. The statistical package SPSS version 24.0 (Statistical Package for Social Sciences Inc., Chicago, IL) and R 3.4.2 software were used for all analyses.

## Results

### *BCR-ABL1*-like signature identification by RNA-Seq

To identify a specific molecular signature for *BCR-ABL1-*like B-ALL, we examined a dataset from patients with B-other ALL (*n* = 49) by targeted RNA-Seq that, after clustering, grouped a subset of patients with overexpression of *CRLF2* and mutations in several genes related to the BCR*-ABL1*-like signature. Thirteen of these patients (27%) were classified as *BCR-ABL1*-like based on heatmap clustering, with all equally showing overexpression of *ENAM* and *IGJ*. Also, most of the patients with high *CRLF2* expression (CRLF2+) were classified into the *BCR-ABL1*-like ALL subgroup: 11/13 (85%) *versus* 7/36 (19%) non-*BCR-ABL1*-like, *P* < 0.001. Mutations of *IKZF1*, *KRAS*, *JAK2*, *CRLF2*, *NRAS*, *JAK1*, *TYK2,* and *PAX5* genes related to *BCR-ABL1*-like genotype were present in 7/11 *BCR-ABL1*-like patients (64%) *versus* 3/20 non-*BCR-ABL1*-like patients (15%), *P* = 0.013. This signature identified *BCR-ABL1*-like patients who showed significantly shorter DFS probability at 5 years [95%confidence interval (CI): 20% (0%; 45%) vs 54% (95% CI: 35%; 73%), *P* = 0.047].

### *BCR-ABL1*-like signature by RNA-Seq, validation cohort

**Fig. 2:**
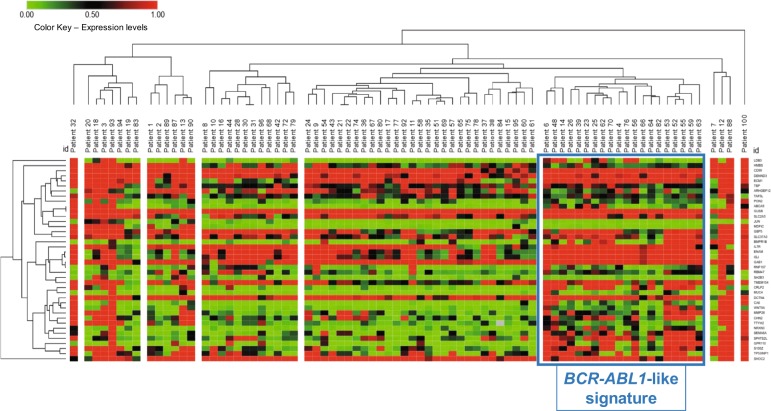
Unsupervised hierarchical cluster with dendrogram of 79 B-other-ALL patients based on gene expression of 40 genes. The 20 *BCR-ABL1*-like patients are marked with a blue square.

We next validated the reproducibility of the identified signature by extending the study cohort to 100 *BCR-ABL1*-negative patients (Fig. 1). Targeted RNA-sequencing was performed on cDNA from 84 patients. Those patients with WHO cytogenetic subtypes (i.e., MLL rearrangements, ploidies, Burkitt ALL), with other hemopathies, or with BCP-ALL with incomplete follow-up data were excluded. A normalized matrix (input for heatmap clustering) was obtained for 79/84 of these patients after removing data for five patients due to the low quality of the generated reads. One of the groups generated in the heatmap was classified as *BCR-ABL1*-like and accounted for 20/79 patients (25%) (Fig. 2). The expression of *ECM1*, *ENAM*, *IGJ,* and *CRLF2* was significantly higher in the *BCR-ABL1*-like subgroup than in the non*-BCR-ABL1*-like subgroup (*P* ≤ 0.015). By contrast, *MDFIC* was underexpressed in 100% of patients with the *BCR-ABL1*-like signature (*P* = 0.001). In addition, this group was enriched inpatients who were CRLF2+ [15/20 (75%) vs 16/59 (27%), *P* < 0.001]; and in mutations related to the BCR*-ABL1*-like signature^16^ such as *JAK2* and *RAS*, or *PAX5* and *IKZF1* genes [14/18 (78%) vs 16/42 (38%), *P* = 0.005]; and both alterations [13/18(72%) vs 6/42 (14%), *P* < 0.001]. Most of the *BCR-ABL1-like* patients agree on the result of *CRLF2* overexpression (17/20: 85%) by both techniques qPCR and RNA-Seq.

### Clinical characteristics and prognosis of patients with *BCR-ABL1*-like ALL

Outcome analysis was performed in patients between 15 and 60 years of age treated in PETHEMA trials with intermediate (10/56, 18%) and high-risk (46/56, 82%) protocols, in order to ensure homogeneity between treatments. With a median follow up of 3.8 years [range, 0.82–14.94], the 4- and 8-year OS of the 56 patients with BCP ALL were 58% (95% CI: 44%; 72%) and 37% (95% CI: 17%; 57%), respectively; the 4- and 8-year DFS were 47% (95% CI: 32%; 62%) and 39% (95% CI: 20%; 58%), respectively; and the CIR was 45% (95% CI: 30%; 59%) for both time points. A summary of the clinical data is shown in Table 1.

Sixteen of the 56 patients (29%) were classified as *BCR-ABL1*-like in the heatmap (Supplementary Fig. 1).

There were no significant differences in baseline characteristics such as age, gender, ALL phenotype, % BM blasts, cytogenetics, and risk classification in *BCR-ABL1*-like patients in comparison with the remaining patients in the B-other ALL subgroup. However, significant differences were observed in the median percentage of blasts in PB between *BCR-ABL1*-like and non-*BCR-ABL1*-like groups (72% vs 34%, *P* = 0.002), and WBC counts [13.40 × 10^9^/L (95% CI: 1.9; 388.0) vs 5.01 × 10^9^/L (95% CI: 0.6; 393.3), *P* = 0.071].

**Fig. 3:**
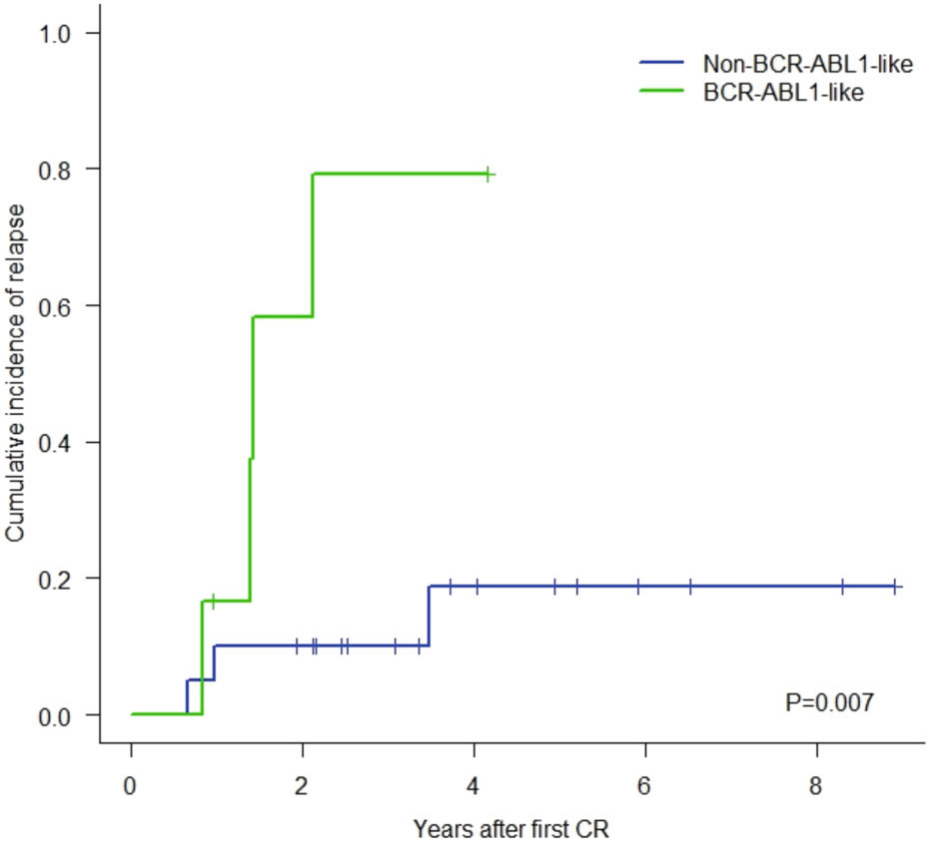
Cumulative incidence of relapse for *BCR-ABL1*-like and non-*BCR-ABL1*-like patients within the post-induction negative-MRD population. *BCR-ABL1*-like patients are denoted by the green line and non-*BCR-ABL1*-like patients by the blue line.

Although there were no significant differences in CR achievement, *BCR-ABL1*-like patients had a tendency for a poorer response to induction treatment than non-*BCR-ABL1*-like patients [3/16 (19%) non-responders in *BCR-ABL1*-like vs 2/40 (5%) in non-*BCR-ABL1*-like patients, *P* = 0.135]. In addition, *BCR-ABL1*-like patients showed a higher proportion of positive MRD than non-*BCR-ABL1*-like patients [8/11 (73%) vs 10/30 (33%), *P* = 0.036] at the end of induction phase. Interestingly, among patients with postinduction negative MRD (MRD-) (*n* = 26), *BCR-ABL1*-like patients (*n* = 6) had a higher rate of relapse and lower CR duration than non-*BCR-ABL1*-like patients (*n* = 20) (*P* = 0.007) (Fig. 3). Nine out of 51 CR patientsunderwent allogeneic-HSCT with no significant differences in the proportion of transplanted patients between *BCR-ABL1*-like and the remaining B-other ALL patients [2/13(15%) vs 7/38 (18%), respectively, *P* = 1.000].

**Fig. 4:**
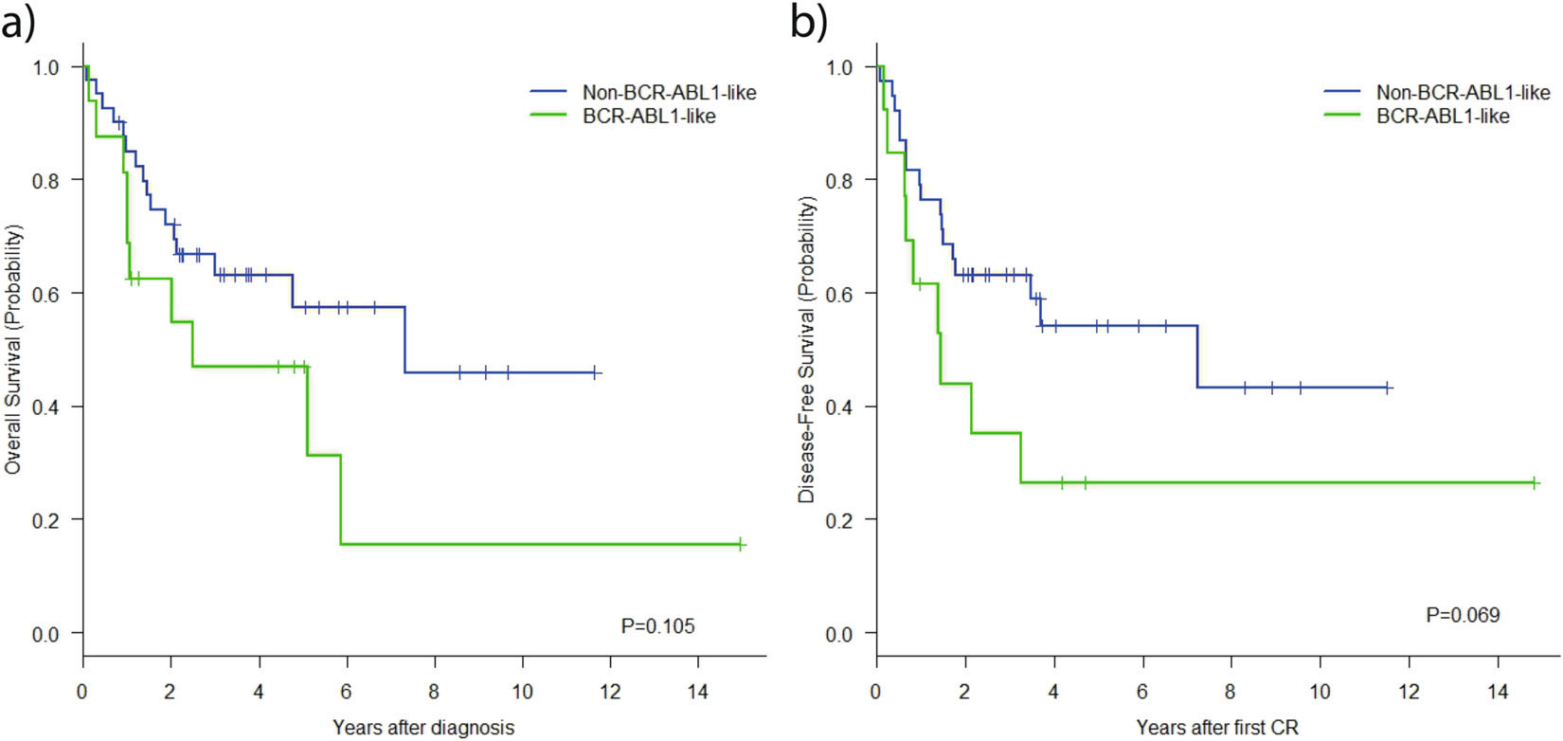
Kaplan–Meier survival curves for BCP-ALL patients: *BCR-ABL1*-like patients are denoted by the green line and non-*BCR-ABL1*-like patients by the blue line. **a** Overall survival; **b** disease-free survival.

*BCR-ABL1*-like patients showed poorer OS than the remaining BCP-ALL patients [47% (95% CI: 21%; 73%) vs 63% (95% CI: 48%; 78%), *P* = 0.105], with a higher frequency of relapses [66% (95% CI: 29%; 87%) for *BCR-ABL1*-like vs 38%(95% CI: 21%; 55%) for B-other ALL, *P* = 0.067] and the consequent lower DFS [26% (95% CI: 1%; 51%) vs 54% (95% CI: 36%; 72%), *P* = 0.069] as the main reasonsfor the poorer OS (Fig. 4). The limited number of patients in this series and the relatively low ratio of mutations did not allow us to assess the possible prognostic significance of JAK/STAT pathway mutations, *N/KRAS*, *IKZF1*, or *PAX5* point mutations. The univariable and multivariable models for the 56 patients are shown in Supplementary Tables S2 and S3.

### *CRLF2* overexpression: association with *BCR-ABL1*-like, JAK/STAT mutation pathway and prognosis

**Fig. 5:**
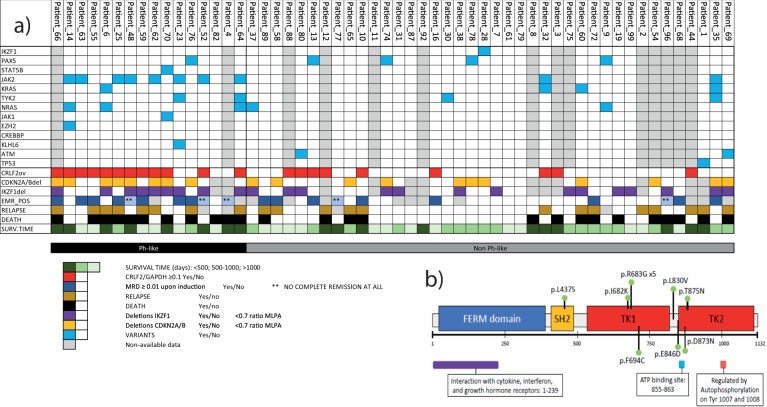
Main alterations identified in this study with a detail of JAK2 mutations. **a** Molecular, genetic, and clinical alterations in *BCR-ABL1*-like and non-*BCR-ABL1*-like patients. **b** Schematic representation of JAK2 primary sequence. Point mutations encountered in the studied population are depicted in green; p.R683G was observed in five patients.

Among the 56 B-other ALL patients with clinical data, most of the *BCR-ABL1*-like patients showed *CRLF2* overexpression clustered in the *BCR-ABL1*-like subgroup [12/16(75%) vs 9/40(22%) non-*BCR-ABL1*-like patients, *P* < 0.001]. There were available samples for NGS molecular studies of DNA for 42/56 patients (14/16 *BCR-ABL1*-like and 24/40 non-*BCR-ABL1*-like). A total of 57 non-synonymous variants affecting 15 genes were identified among the 42 patients. At least one variant could be identified in 79% (33/42) of the patients, and at least one pathogenic mutation was identified in 57% (24/42). The distribution of the mutated genes is shown in Fig. 5a and in more detail in Supplementary Table S4. JAK2 mutations (more recurrently c.2047A > G and p.R683G) were enriched in *BCR-ABL1*-like ALL patients [9/14(64%) vs non-BCR*-ABL1*-like 3/28(11%), *P* = 0.001]. The distribution of JAK2 mutations across the protein domains is shown in Fig. 5b.

Significant differences in overexpression of *CRLF2* were found between *BCR-ABL1*-like and non-*BCR-ABL1*-like groups (see Supplementary Table S5, *P* < 0.001), and additional mutations were found in JAK-STAT, RAS, and transcription factors such as IKZF1 or PAX5. Statistically significant differences were found in the rate of JAK/STAT mutations between *BCR-ABL1*-like patients and the remaining B-other ALL patients [*BCR-ABL1*-like 9/14 (64%) vs 3/28 (11%), *P* = 0.001], but not for RAS genes or lymphoid transcription factors.

We found significant differences in DFS for CRLF2 overexpression [4-year DFS 28% (95% CI: 6%; 50%) vs 59% (95% CI: 40%; 78%) and 8-year DFS 28% (95% CI: 6%; 50%) vs 47% (95% CI: 22%; 72%), *P* = 0.006] (Supplementary Fig. 2b). CRLF2+ patients had higher 4- and 8-year CIR than patients with no CRLF2 overexpression [61% (95% CI: 33%; 81%) vs 35% (95% CI: 17%; 54%), *P* = 0.018; at both time points] (Supplementary Fig. 2c). Although CRLF2+ patients showed lower OS than those without overexpression, the differences were not statistically significant [4-year OS 52% (95% CI: 29%; 75%) vs 62% (95% CI: 46%; 78%) and 8-year OS 20% (95% CI: 0%; 51%) vs 45% (95% CI: 20%; 70%), *P* = 0.234].

Of note, no differences were observed in CR achievement, MRD clearance or outcome between CRLF2+*/BCR-ABL1*-like and CRLF2+/non-*BCR-ABL1*-like patients (data not shown).

### *Prognostic impact of other molecular alterations: IKZF1 and CDKN2A/B deletions*

Analysis of copy number alterations by MLPA was available for 44/56 B-other ALL patients. Results showed that 75% of these patients (33/44) had at least one deletion in *IKZF1* or *CDKN2A/B*, but no significant differences were found between *BCR-ABL1*-like and non-*BCR-ABL1*-like groups, or for individual deletions in the case of codeletion of both genes (Table 1).

As shown in univariable and multivariable analysis (Supplementary Tables S2 and S3), *CDKN2A/B* deletions showed statistical significance for outcome prediction in the 56 B-other ALL patients. However, we observed significant differences regarding the impact of these deletions within *BCR-ABL1*-like and non-*BCR-ABL1*-like subgroups individually. Although the number of patients was low, *BCR-ABL1*-like patients with *CDKN2A/B* deletion (*n* = 6) showed lower OS than equivalent patients without loss of *CDKN2A/B* (*n* = 8) [4-year OS 17% (95% CI: 0%; 46%) vs 83% (95% CI: 53%; 100%), *P* = 0.041]. However, these differences showed a trend for DFS and were not statistically significant for CIR, in which patients with *CDKN2A/B* loss had higher relapse incidence in both subgroups individually. By contrast, the prognostic differences on OS and DFS observed for *BCR-ABL1*-like were not observed within the non-*BCR-ABL1*-like patients, although non-*BCR-ABL1*-like patients with *CDKN2A/B* deletion showed a trend towards higher CIR than those without *CDKN2A/B* loss (data not shown).

*IKZF1* deletions did not have prognostic significance for any outcome parameter analyzed in the B-other ALL series. However, the concomitance of *IKZF1* and *CDKN2A/B* deletions identified a subset of patients with poor prognosis. Among the 44 patients with known *IKZF1* and *CDKN2A/B* status, those having both alterations (*n* = 11) showed poorer OS than those without both deletions (*n* = 33) [4-year OS 46% (95% CI: 17%; 72%) vs 67% (95% CI: 50%; 84%), *P* = 0.090]. The same scenario was observed for DFS [3-year DFS 40% (95% CI: 10%; 70%) vs 67% (95% CI: 50%; 84%), *P* = 0.014] and CIR [3-year CIR 50% (95% CI: 16%; 77%) vs 30% (95% CI: 15%; 47%), *P* = 0.061].

**Fig. 6:**
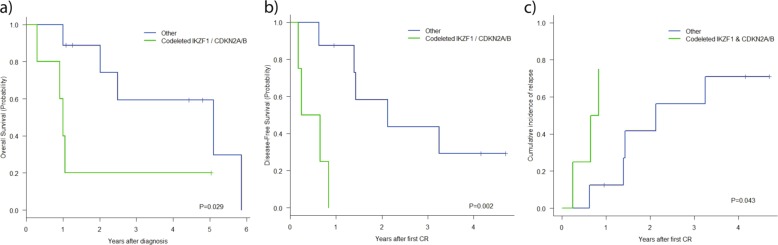
Log-rank comparison curves between *IKZF1* and *CDKN2A-B*-codeletion positive and negative patients within the *BCR-ABL1*-like population. *IKZF1/CDKN2A-B* codeleted patients are denoted by the green line and patients with no codeletion of *IKZF1/CDKN2A-B* by the blue line. **a** Overall survival; **b** disease-free survival; **c** cumulative incidence relapse.

Subgroup analysis was repeated for patients with both deletions, to assess whether the concomitant loss of *IKZF1* and *CDKN2A/B* had adifferent prognosis in *BCR-ABL1*-like and non-*BCR-ABL1*-like cohorts individually. Again, although the patient numbers were low, we observed that within the *BCR-ABL1*-like subset, patients harboring both deletions experienced significantly lower probability of survival than those without both alterations (4/5 vs 5/9 deaths, *P* = 0.029), mainly due to a higher relapse rate (3/4 *i* 5/8 relapses, *P* = 0.043) (Fig. 6). Within the remaining B-other ALL subgroup, patients with deletion of both genes also experienced more relapses although the results were not statistically significant (data not shown).

## Discussion

We sought to identify an RNA-Seq signature for *BCR-ABL1*-like ALL in a series of homogeneously treated adolescent and adult patients with B-other ALL, and analyze its prognostic impact. We demonstrate the capacity of a simplified targeted RNA-Seq signature to segregate *BCR-ABL1*-like patients within a BCP-ALL adult population. This approach enabled us to confirm that *BCR-ABL1*-like is a high-risk ALL subtype also in young, adolescent, and older adults despite treatment with modern MRD-oriented protocols.

The prognostic impact of the *BCR-ABL1*-like subtype has been mainly reported in pediatric and adolescent populations, and studies in adult BCP-ALL are scarce^6,8,16^. The DCOG/Erasmus and St Jude groups used different gene profiles to define the *BCR-ABL1*-like subtype and, consequently, there is a lack of standardization and comparability regarding the best strategy to identify these patients. The frequency (29%) and clinical outcome of *BCR-ABL1*-like ALL found in this study is in accord with that of the MD Anderson and St. Judereports^9,11^: Roberts and coworkers^11^ characterized 194/798 (24%) patients (age range 21–86 years) as *BCR-ABL1*-like, and Jain et al.^9^ identified 49/148 (33%) patients (age range 15–71 years). Regarding *IKZF1* deletions, the MD Anderson group classified 73% of patients within the BCR*-ABL1*-like population as positive, St. Jude reported 81% of *IKZF1* losses, and we found 64% of mutated patients. In our study, we identified 75% of patients CRLF2+ and 57% with mutated *JAK2*, similar to the published results. Indeed, 61% and 51% of CRLF2+ and 45% and 27% of mutated JAK2 have been reported within *BCR-ABL1*-like patients in the St. Jude and MD Anderson studies, respectively.

The prognostic impact of the *BCR-ABL1*-like ALL subtype in adults homogeneously treated within MRD-oriented trials remains still unclear. In our series, these patients had higher WBC and blast counts than non-*BCR-ABL1*-like patients, suggesting a greater degree of cell proliferation with a strong capacity for dissemination—an essential characteristic related to a higher degree of clonal heterogeneity and treatment resistance. Interestingly, a high degree of cell cycle deregulation (57% *CDKN2A/B* deletion) and stem cell-like characteristics as a result of *IKZF1* losses (64%) might play an important role in the aggressiveness and resistance seen in the present and other series of *BCR-ABL1*-like patients. As mentioned, 75% of *BCR-ABL1*-like patients overexpressed *CRLF2* and 64% had mutations in the JAK-STAT pathway (and all CRLF2+ patients also had *JAK2* mutations), supporting the idea that this is an essential pathway in this aggressive subtype. Most JAK2 mutations identified in the present study were pathogenic and involved the tyrosine-kinase domains 1 and 2, close to the nucleotide binding site for ATP-ADP exchange (residues 855–863), and structurally distant from the self-regulatory tyrosine 1007 and 1008. Currently, several clinical trials are evaluating the efficacy of JAK and mTOR inhibitors (in addition to TKI inhibitors), alone or in combination with other drugs based on this genetic rationale^24–26^.

*IKZF1* and *CDKN2A/B* deletions are known to be associated with poor prognosis in pediatric and adult B-ALL populations^27–29^, mainly in the *BCR-ABL1* B-ALL subpopulation. Our results suggest that, despite the low number of cases analyzed, *CDKN2A/B* deletions are markers of poor survival in B-other ALL, and the association between *CDKN2A/B* and *IKZF1* deletions might also contribute to the dismal prognosis of *BCR-ABL1*-like. Finally, the survival analysis suggests that all CRLF2+ patients show poor response to standard treatment and bad prognosis whether or not they are *BCR-ABL1*-like. Unfortunately, we did not have enough samples to evaluate the prognostic significance of additional alterations (i.e., *JAK2* mutations, *IKZF1,* and *CDKN2A/B* deletions) in the cohort of 56 B-other ALL patients or within the subgroups of patients with and without *CRLF2* overexpression.

Due to the limited number of *BCR-ABL1*-like patients in our series, the difference in CR achievement between *BCR-ABL1*-like and non-*BCR-ABL1*-like patients did not reach statistical significance, although there were four times as many patients not achieving CR in the *BCR-ABL1*-like subgroup. Taken together with the low MRD clearance at the end of induction seen in *BCR-ABL1*-like patients (three-quarters of *BCR-ABL1*-like patients were MRD-positive at the end of induction compared with one-third of non-BCR*-ABL1*-like patients), this indicates higher treatment resistance. Given the small number of B-other patients (*n* = 10) MRD-positive at the end of consolidation, we could not evaluate the role of HSCT on *BCR-ABL1*-like and non-*BCR-ABL1*-like patients.

In addition to treatment resistance, the high degree of relapse observed in *BCR-ABL1*-like patients and, more importantly, those who were MRD-negative at the end of induction, suggests that standard treatments are not less efficient for this ALL subtype. Also, while MRD has become the most powerful outcome predictor in ALL, it is not fully predictive, and other factors beyond MRD (e.g., genetic alterations) also impact patients’ prognosis, especially in the B-other subpopulation.

The *BCR-ABL1*-like signature methodology clearly distinguishes the outcome of patients in whom no recurrent genetic abnormalities could be identified by standard methods. Specifically, loss of *CDKN2A/B* identifies patients at high risk of disease progression among those with non-available genetic risk categorization. We also provide more detailed information on the prognostic impact of *IKZF1* and *CDKN2A/B* deletions (together and separately), specifically within the *BCR-ABL1*-like subtype, where they seem to confer poor outcome. By contrast, the prognostic relevance of these alterations in the non-*BCR-ABL1*-like subgroup is less clear. Due to the paucity of samples, more studies are needed to address these issues.

Currently, methodical characterization *BCR-ABL1*-like is expensive and laborious. The multiple rearrangements present in this subtype and the constellation of other molecular alterations characteristic but not exclusive of this entity make NGS an attractive option, although this requires complex bioinformatic analysis. Interestingly, the approach shown here enables the identification of the *BCR-ABL1*-like signature by targeted RNA-Seq in 3 days upon sample arrival with a simple and fast NGS library protocol and subsequent sequencing. It is also reproducible, as we have shown in the validation cohort. Finally, a simple computer plug-in will output a normalized matrix that will predict patient outcome in the hierarchical clustering heatmap.

In summary, our study demonstrates that targeted RNA-Seq correctly identifies *BCR-ABL1*-like ALL in BCP-ALL patients. This methodology is inexpensive, available, and feasible, and could be introduced in the routine clinical workout for ALL patients. We have also confirmed the poor prognosis of *BCR-ABL1*-like ALL in the adult setting. An early diagnosis of *BCR-ABL1*-like ALL could be critical to initiate appropriate treatment depending on the kinase profile (i.e., JAK inhibitor, tyrosine-kinase inhibitor, etc.). These results endorse the inclusion of these molecular approaches in further clinical protocols for adopting early clinical decisions with the goal to better manage patients with ALL.

Supplementary information is available at *Blood Cancer Journal* website.

## Supplementary information

Supplementary Tables S1, S2, S3, and S5

Supplementary Table S4

Figure S1 and Figure S2
