# Supplementary material for: A novel targeted RNA-Seq panel identifies a subset of adult patients with acute lymphoblastic leukemia with BCR-ABL1-like characteristics
Source: Blood Cancer J. 2020 Apr 24;10(4):43. doi: 10.1038/s41408-020-0308-3 (PMC7182567; doi:10.1038/s41408-020-0308-3)
Supplement: Supplementary file 1 — Supplementary Tables S1, S2, S3, and S5 [file 41408_2020_308_MOESM1_ESM.pdf]

Supplementary Information

A novel targeted RNA-Seq panel identifies a subset of adult patients with acute lymphoblastic leukemia with *BCR-ABL1*-like characteristics

Ricardo Sánchez[1,2,3≠], PhD, Jordi Ribera[4], PhD, Mireia Morgades[4], MS, Rosa Ayala[1,2,3,5,6], PhD, Esther Onecha[1,2], PhD, Yanira Ruiz-Heredia[1], PhD, Alexandra Juárez-Rufián[1], MS, Rodrigo de Nicolás[1], MS, José Sánchez-Pina[1], MD, Susana Vives[4], PhD, Lurdes Zamora[4], PhD, Santiago Mercadal[7], PhD, Rosa Coll,[8], PhD, Marta Cervera[9], PhD, Olga García[4], MD, Josep-Maria Ribera[4,*], PhD and Joaquín Martínez-López[1,2,3,4,5 *,≠], PhD

1 – Hematology Department, Hospital Universitario 12 de Octubre, Madrid;
2 – Instituto de Investigación Hospital 12 Octubre (i+12)
3 – Hematological Malignancies Clinical Research Unit, CNIO, Madrid;
4 – Servicio de Hematología, ICO- Hospital Germans Trias i Pujol. Josep Carreras Leukaemia Research Institute, Universidad Autònoma de Barcelona, Badalona, Spain
5 - Centro de Investigación Biomédica en Red Cáncer (CIBERONC), Madrid;
6 – Universidad Complutense de Madrid (UCM), Madrid;
7 – Hematology Department, ICO – Hospital Duran i Reynals (Bellvitge), Barcelona. Spain
8 – Hematology Department, ICO – Hospital Dr. Josep Trueta, Girona. Spain
9 – Hematology Department, ICO – Hospital Universitari Joan XXIII, Tarragona. Spain

*These authors contributed equally to this work

Supplemental Information Text Summary: two supplemental figures (Powerpoint file) with figure legends, and five supplemental tables (one of them as a separate Excel file).

Supplemental Figure Legends:

Supplementary Figure S1. Unsupervised hierarchical cluster with dendrogram of 56 BCP-ALL patients between 15 and 60 years old, based on gene expression of 40 genes. The 16 *BCR-ABL1*-like patients are blue squared.

Supplementary Figure S2. Log-rank comparison curves of molecular *CRLF2* overexpression patients *Positive* Vs *Negative*. *CRLF2* overexpressed patients are denoted by the green line and patients with no *CRLF2* overexpression by the blue line; a) Overall Survival, b) Disease-free survival, and c) Cumulative incidence of relapse.

Supplementary Tables:

Supplementary Table S1. Detailed main characteristics of the patients.

Patient ID	WBC≠ ·10^{-9} (L)	Sex	Age≠	CRLF2 / GAPDH expression %	DNA-Seq mutations	Date of Diagnostic	OS (months)	BM blasts (%)	ALL Type	Treatment Protocol	Complete Remission	Start Date of Treatment	Response Date	HSCT (1st CR)	Relapse	Death	Last Follow-up Date
1	3.00	F	57	<0.01%	No	08/15/05	10.98	N/A	Common	HR ALL-2003	Yes	08/31/05	10/05/05	No	Yes	Yes	07/15/06
2	2.50	F	52	<0.01%	N/A	08/24/06	57.11	79%	Common	HR ALL-2003	Yes	08/30/06	10/03/06	No	Yes	Yes	05/27/11
3	2.86	F	41	0.20%	N/A	05/26/09	8.38	100%	Common	HR ALL-2003	Yes	05/26/09	06/07/09	No	No	Yes	02/05/10
4*	2.1	M	35	<0.01%	N/A	10/20/10	1.51	95%	Common	HR ALL-2003	No	10/21/10	N/A	N/A	N/A	Yes	12/05/10
6*	190	F	22	13.56%	Yes	07/20/15	12.00	95%	Common	HR ALL-2011	Yes	07/21/15	08/25/15	No	Yes	Yes	07/19/16
7	23.0	M	39	<0.01%	No	08/06/15	45.73	94%	Common	HR ALL-2011	Yes	08/07/15	09/07/15	No	No	No	05/28/19
8	0.90	M	40	<0.01%	N/A	05/16/13	5.26	75%	Common	HR ALL-2011	Yes	05/17/13	06/20/13	No	No	Yes	10/23/13
9	3.40	M	48	0.03%		07/20/15	44.55	85%	Common	HR ALL-2011	Yes	07/23/15	09/01/15	No	No	No	04/05/19
10	393	M	27	0.12%	No	04/26/16	14.50	95%	Pro-B	HR ALL-2011	Yes	04/29/16	06/07/16	Yes	Yes	Yes	07/11/17
11	4.70	F	27	0.01%	N/A	10/28/13	49.81	80%	Common	IR ALL-2008	Yes	11/04/13	12/10/13	No	No	No	12/21/17
12	11.7	F	22	16.36%	N/A	03/09/16	11.7	N/A	Common	1. IR ALL-2008;<br>2. HR ALL-2003	Yes	03/11/16	04/21/16	No	Yes	Yes	02/28/17
13	2.00	M	20	0.12%	Yes	06/10/16	27.12	80%	Common	IR ALL-2008	Yes	06/15/16	07/26/16	No	No	No	09/13/18
14*	2.00	M	18	4.44%	Yes	02/24/17	12.99	93%	Common	IR ALL-2008	Yes	02/28/17	04/11/17	No	No	No	03/26/18
16	9.70	F	32	0.12%	No	09/15/10	102.87	N/A	Pre-B	HR ALL-2003	Yes	N/A	12/24/10	No	No	No	04/10/19
19	N/A	F	48	0.01%	No	12/15/12	18.54	94%	Common	HR ALL-2011	Yes	N/A	01/21/13	Yes	No	Yes	07/02/14
23*	3.51	M	39	0.08%	Yes	11/15/13	53.1	98%	Pre-B	HR ALL-2011	Yes	12/02/13	02/20/14	No	No	No	04/18/18
25*	10.9	M	22	5.11%	Yes	09/10/14	29.82	98%	Pre-B	IR ALL-2008	No	09/12/14	12/01/14	No	Yes	Yes	03/05/17
28	2.20	M	38	<0.01%	Yes	02/08/16	27.06	93%	Common	HR ALL-2003	Yes	N/A	03/15/16	No	No	No	05/11/18
30	N/A	M	34	<0.01%	Yes	03/31/16	27.16	N/A	Common	HR ALL-2011	Yes	04/06/16	06/10/16	Yes	No	No	07/05/18

31	N/A	F	28	<0.01%	No	04/18/16	37.51	99%	Common	1. IR ALL-2008; 2. HR ALL-2003	Yes	N/A	07/07/16	No	No	No	06/03/19
32	N/A	M	40	6.11%	Yes	06/29/16	9.86	N/A	Common	HR ALL-2011	Yes	N/A	08/16/16	No	Yes	No	04/25/17
35	22.0	M	57	0.04%	Yes	10/05/16	26.17	>90%	Common	HR ALL-2011	Yes	10/11/16	01/03/17	No	No	No	12/10/18
37	2.90	F	23	<0.01%	Yes	11/02/16	30.81	93%	Common	HR ALL-2011	Yes	11/07/16	12/12/16	No	No	No	05/28/19
38	3.70	F	34	<0.01%	No	11/06/16	31.63	91%	Common	HR ALL-2011	Yes	11/17/16	12/16/16	No	No	No	06/26/19
44	2.68	F	55	1.44%	N/A	06/01/17	24.89	60%	Common	HR ALL-2011	Yes	06/05/17	07/15/17	Yes	Yes	No	06/27/19
48*	61.4	F	22	11.22%	Yes	08/24/17	10.92	Dry aspirate	Common	HR ALL-2011	No	08/30/17	N/A	No	No	Yes	07/22/18
52*	45.0	F	48	2.65%	Yes	07/09/07	15.02	N/A	Pre-B	HR ALL-2003	No	N/A	N/A	N/A	N/A	No	10/08/08
54	5.50	M	52	<0.01%	N/A	03/30/09	22.52	N/A	Common	HR ALL-2003	Yes	N/A	04/28/09	No	Yes	Yes	02/13/11
55*	58.3	F	21	3.85%	Yes	05/26/10	70.32	N/A	Common	HR ALL-2003	Yes	N/A	06/28/10	No	Yes	Yes	04/03/16
58	14.6	M	24	<0.01%	No	12/07/11	72.23	N/A	Common	1.IR ALL-2008; 2.HR ALL-2003	Yes	N/A	01/17/12	Yes	No	No	12/12/17
59*	47.8	F	22	7.38%	No	12/15/09	61.15	N/A	Common	HR ALL-2003	Yes	N/A	01/21/10	No	Yes	Yes	01/18/15
60	196	F	46	0.02%	Yes	03/21/06	16.44	N/A	Common	HR ALL-2003	Yes	N/A	04/05/06	No	Yes	Yes	08/03/07
61	7.40	M	39	0.06%	No	04/12/10	38.27	N/A	Common	HR ALL-2003	Yes	N/A	05/21/10	No	No	No	06/19/13
62*	43.0	M	29	4.01%	Yes	12/09/10	60.43	N/A	Common	HR ALL-2003	Yes	N/A	01/20/11	No	Yes	No	12/21/15
63*	12.3	M	19	81.91%	No	06/14/11	57.60	N/A	Pro-B	1.IR ALL-2008; 2.HR ALL-2003	Yes	N/A	07/21/11	Yes	No	No	03/31/16
64*	14.5	M	59	3.90%	Yes	09/27/10	12.03	N/A	Pre-B	HR ALL-2003	Yes	N/A	11/03/10	Yes	Yes	Yes	09/28/11
65	48.0	F	25	0.01%	No	11/16/09	24.95	N/A	Pro-B	HR ALL-2003	Yes	N/A	12/28/09	No	Yes	Yes	12/15/11
66*	388	M	17	1.51%	N/A	08/13/07	12.66	N/A	Common	HR ALL-2003	Yes	N/A	09/12/07	No	Yes	Yes	09/01/08
68	9.00	F	54	<0.01%	N/A	01/26/05	88.01	N/A	Common	HR ALL-2003	Yes	N/A	03/02/05	No	No	Yes	05/26/12
69	9.60	M	58	<0.01%	No	04/29/09	25.61	N/A	Common	HR ALL-2003	Yes	N/A	06/04/09	No	Yes	Yes	06/17/11
70*	9.80	F	38	16.78%	Yes	04/28/12	3.55	N/A	Common	HR ALL-2011	Yes	N/A	06/12/12	No	No	Yes	08/14/12
72	9.60	F	46	<0.01%	No	06/08/09	17.36	N/A	Common	HR ALL-2003	Yes	N/A	07/15/09	No	Yes	Yes	11/18/10
74	2.20	F	27	<0.01%	Yes	08/20/13	69.76	N/A	Pro-B	IR ALL-2008	Yes	N/A	09/30/13	No	Yes	No	06/12/19
75	8.33	F	41	0.01%	N/A	03/25/14	60.49	N/A	Pro-B	HR ALL-2011	Yes	N/A	04/28/14	No	No	No	04/08/19
76*	1.90	M	44	0.03%	Yes	02/28/12	24.23	N/A	Common	HR ALL-2011	Yes	N/A	04/11/12	No	Yes	Yes	03/06/14
77	12.9	F	20	0.01%	No	09/16/11	0.79	N/A	Pro-B	IR ALL-2008	No	N/A	N/A	N/A	N/A	Yes	10/10/11

78	0.60	M	34	0.05%	Yes	08/29/14	41.52	N/A	Pre-B	HR ALL-2011	Yes	N/A	10/06/14	No	No	No	02/12/18
79	1.50	M	39	0.02%	No	05/12/10	109.71	N/A	Common	HR ALL-2003	Yes	N/A	08/09/10	No	No	No	07/01/19
80	2.10	F	17	0.11%	No	02/22/12	64.47	N/A	Common	IR ALL-2008	Yes	N/A	04/24/12	No	No	No	07/06/17
82*	6.37	M	21	<0.01%	No	10/22/02	179.31	98%	Pre-B	IR ALL 96	Yes	10/26/02	12/10/02	No	No	No	09/27/17
87	2.52	F	31	<0.01%	No	10/19/07	139.69	Dry aspirate	Pre-B	HR ALL-2003	Yes	11/14/07	12/19/07	No	No	No	06/15/19
88	18.1	M	16	0.63%	N/A	06/05/08	45.11	95%	Pre-B	IR ALL-2008	Yes	06/06/08	07/10/08	Yes	No	No	03/08/12
89	4.71	F	23	<0.01%		02/18/09	36.07	N/A	Pre-B	IR ALL-2008	Yes	02/26/09	03/27/09	Yes	Yes	Yes	02/20/12
92	90.0	F	31	0.04%	N/A	10/19/09	115.92	Dry aspirate	Pre-B	HR ALL-2003	Yes	10/28/09	12/02/09	No	No	No	06/15/19
96	3.25	F	53	<0.01%	N/A	02/25/11	3.48	N/A	Pre-B	HR ALL-2003	No	03/09/11	N/A	No	No	Yes	06/11/11
99	5.30	M	49	<0.01%	No	05/30/12	79.36	92%	Pro-B	HR ALL-2011	Yes	06/04/12	07/03/12	No	No	No	01/08/19

(≠) At diagnosis; (*) *BCR-ABL1*-like; CR, Complete Response; HR, High Risk; IR, Intermediate Risk; N/A, Non-available data

Supplementary Table S2. Univariant analysis of the risk factors affecting BCP-ALL population.

**Variable**		**OS post-CR, HR (CI 95%)**	**P**	**DFS, HR (CI 95%)**	**P**	**CIR, HR (CI 95%)**	**P**
**Age**	(continuous)	1.029 (0.995; 1.064)	0.099	1.025 (0.994; 1.057)	0.120	1.013 (0.975; 1.052)	0.510
**Age**	>35 years	2.270 (0.964; 5.345)	0.061	1.838 (0.843; 4.009)	0.126	1.019 (0.441; 2.355)	0.970
**WBC**	(continuous)	1.006 (1.002; 1.010)	**0.004**	1.005 (1.001; 1.009)	**0.006**	1.007 (1.004; 1.010)	**<0.001**
**Gender**	Female	1.687 (0.704; 4.044)	0.241	1.746 (0.790; 3.858)	0.168	0.718 (0.309; 1.667)	0.440
**PB blasts (%)**	(continuous)	1.011 (0.996; 1.026)	0.168	1.014 (0.999; 1.028)	0.062	1.021 (1.003; 1.039)	**0.021**
**BM blasts (%)**	(continuous)	1.009 (0.923; 1.103)	0.841	0.953 (0.888; 1.023)	0.185	0.948 (0.873; 1.028)	0.200
**Protocol**	High risk	2.557 (0.596; 10.964)	0.206	2.145 (0.643; 7.157)	0.214	0.646 (0.225; 1.849)	0.410
***BCR-ABL1*-like**	Positive	1.889 (0.790; 4.516)	0.153	2.087 (0.926; 4.703)	0.076	2.290 (0.970; 5.407)	0.059
CRLF2/GAPDH	Positive	1.989 (0.853; 4.634)	0.111	2.850 (1.310; 6.201)	**0.008**	2.774 (1.200; 6.412)	**0.017**
**JAK/STAT**	Mutation	1.198 (0.329; 4.366)	0.784	1.595 (0.566; 4.493)	0.377	1.215 (0.384; 3.842)	0.740
N/KRAS	Mutation	2.151 (0.577; 8.014)	0.254	1.969 (0.635; 6.108)	0.241	2.515 (0.717; 8.829)	0.150
IKZF1* or *PAX5	Mutation	0.372 (0.049; 2.852)	0.341	0.552 (0.127; 2.409)	0.429	0.663 (0.179; 2.460)	0.540
IKZF1	Deletion	1.012 (0.386; 2.654)	0.980	1.177 (0.474; 2.920)	0.725	0.918 (0.350; 2.411)	0.860
CDKN2A/B	Deletion	2.523 (0.916; 6.947)	0.073	2.861 (1.092; 7.497)	**0.032**	2.900 (1.057; 7.953)	**0.039**
IKZF1* & *CDKN2A/B	Codeletion	2.246 (0.760; 6.632)	0.143	3.148 (1.205; 8.227)	**0.019**	2.580 (0.983; 6.772)	**0.054**
**MRD (end induction)**	>0.01%	2.001 (0.774; 5.172)	0.152	1.948 (0.818; 4.639)	0.132	1.855 (0.704; 4.887)	0.210

BM, Bone Marrow; CI, Confidence Interval; CIR, Cumulative Incidence of Relapse; CR, Complete Remission; DFS, Disease-free Survival; HR, Hazard Ratio; OS, Overall Survival; PB, Peripheral Blood; WBC, White Blood Cells

Supplementary Table S3. Multivariate analysis of the main factors affecting BCP-ALL patients prognosis.

		OS post-CR		DFS		CIR	
**Variable**		**OS post-CR, HR (CI 95%)**	**P**	**DFS, HR (CI 95%)**	**P**	**CIR, HR (CI 95%)**	**P**
**Age**	(continuous)	-	0.128	Not included		Not included	
**WBC**	(continuous)	1.010 (1.003; 1.017)	**0.003**	1.010 (1.004; 1.017)	**0.001**	1.017 (1.001; 1.033)	**0.043**
CDKN2A/B	Deletion	4.039 (1.151; 14.169)	**0.029**	2.940 (0.940; 9.195)	0.064	-	0.140
**MRD (end induction)**	≥0.01%	-	0.959	-	0.359	-	0.770
***BCR-ABL1*-like**	Positive	3.134 (0.948; 10.358)	0.061	-	0.108	-	0.520

CI, Confidence Interval; CIR, Cumulative Incidence of Relapse; CR, Complete Remission; DFS, Disease-free Survival; HR, Hazard Ratio; OS, Overall Survival; WBC, White Blood Cells

Supplementary Table S4. Detailed variants found in DNA Sequencing analysis.

As a separate excel file.

Supplementary Table S5. Summary of the mutational status of patients according to their overexpression of *CRLF2* and classification as *BCR-ABL1*-like.

Pathway		***BCR-ABL1*-like (n=14)**	**Non-*BCR-ABL1*-like (n=28)**	**P-value**	**CRLF2/GAPDH (>0.1%) (n=16)**	**CRLF2/GAPDH (≤0.1%) (n=26)**	**P-value**
**JAK-STAT**	Mutated	9 (64%)	3 (11%)	0.001	9 (56%)	3 (12%)	0.004
	WT	5 (36%)	25 (89%)		7 (44%)	23 (88%)	
**RAS**	Mutated	3 (21%)	5 (18%)	1.000	4 (25%)	4 (15%)	0.454
	WT	11 (79%)	23 (82%)		12 (75%)	22 (85%)	
**KINASES**	Mutated	1 (7%)	5 (18%)	0.645	1 (6%)	5 (19%)	0.380
	WT	13 (93%)	23 (82%)		15 (94%)	21 (81%)	

WT, Wild-type
