# Supplementary figures and images for: A novel targeted RNA-Seq panel identifies a subset of adult patients with acute lymphoblastic leukemia with BCR-ABL1-like characteristics
Source: Blood Cancer J. 2020 Apr 24;10(4):43. doi: 10.1038/s41408-020-0308-3 (PMC7182567; doi:10.1038/s41408-020-0308-3)

## Slide 1
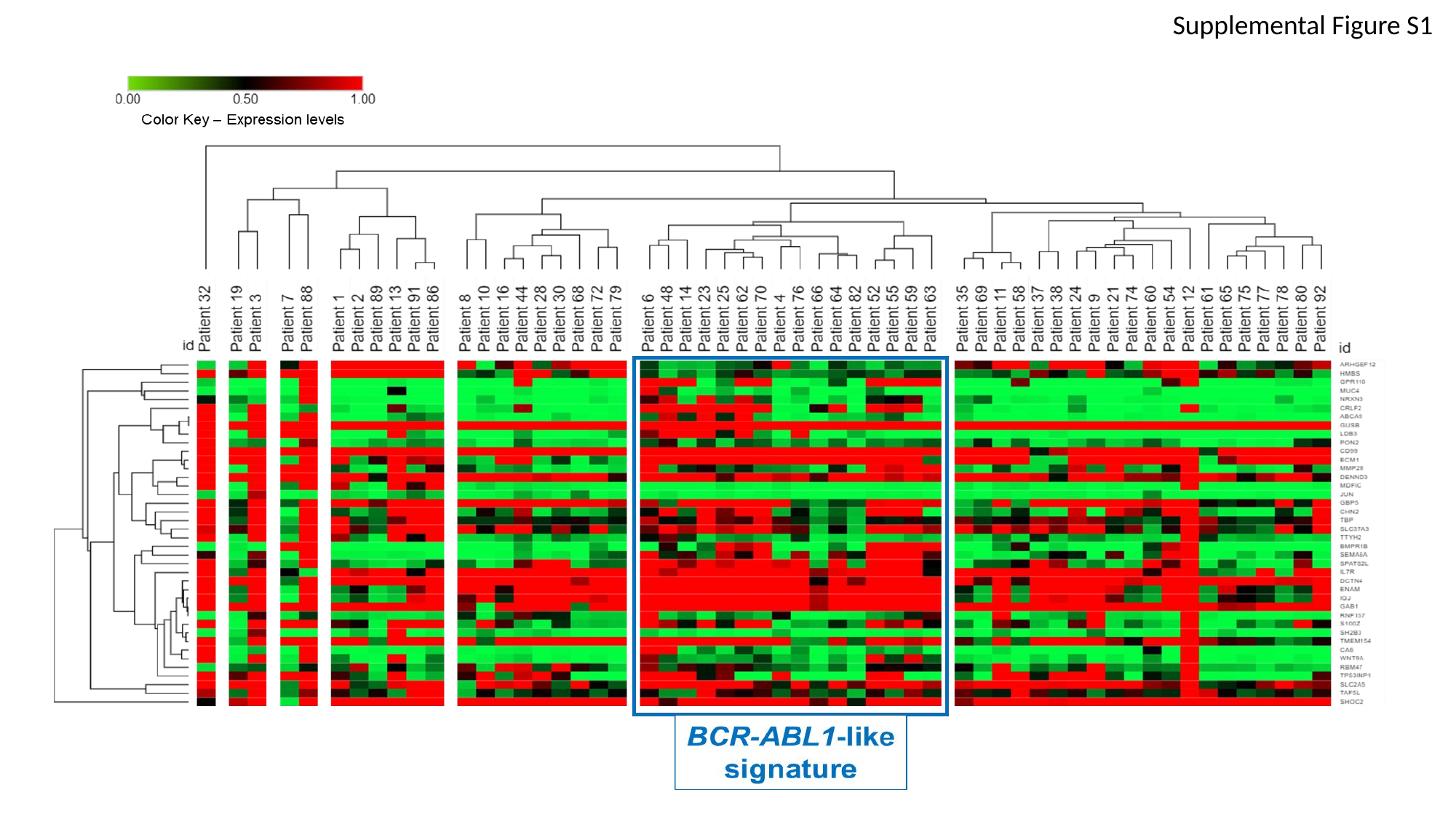

Supplemental Figure S1

## Slide 2
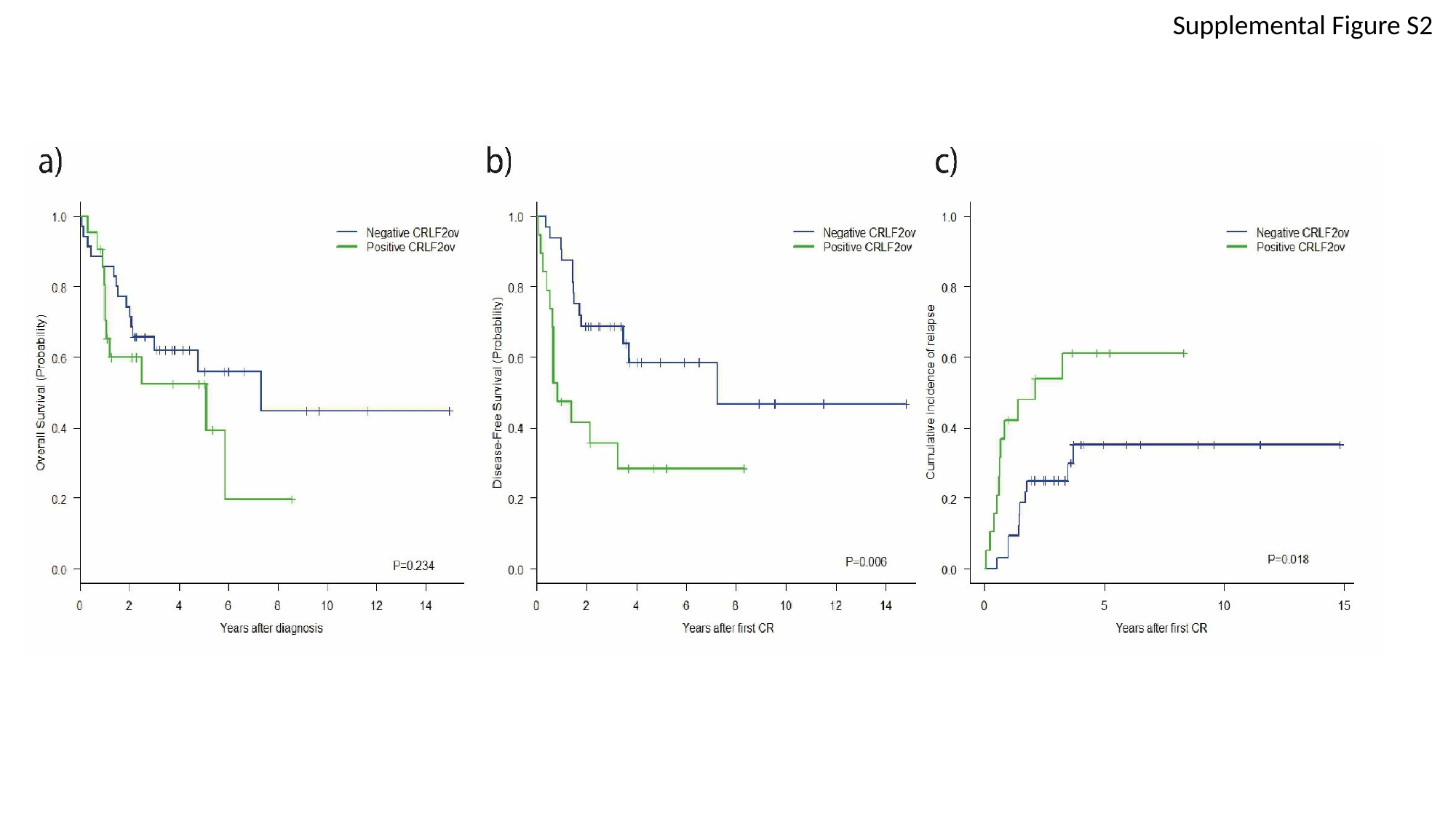

Supplemental Figure S2

Supplement: Supplementary file 3 — Figure S1 and Figure S2 [file 41408_2020_308_MOESM3_ESM.pptx]
